# Supplementary material for: Molluscivorous red knots rapidly adjust to a plant diet
Source: Biol Open. 2026 Apr 15;15(4):bio062365. doi: 10.1242/bio.062365 (PMC13133769; doi:10.1242/bio.062365)
Supplement: Supplementary information [file biolopen-15-062365-s1.pdf]

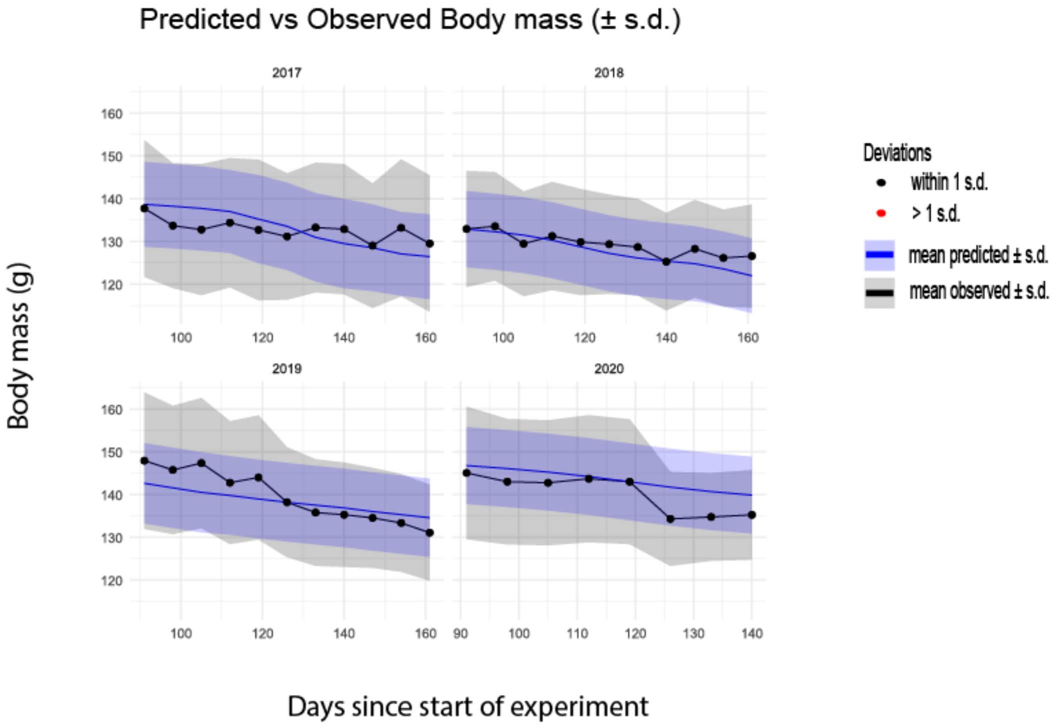


**Fig. S1. Internal model evaluation using the leave-one-out validation (LOOV) method**. Model predictions are made for the same period during which the experimental birds (in the winter of 2023-2024) received a vegetarian diet and onwards. Weekly predictions are compared to the observed data and all deviations of the mean is less than 1 standard deviation (black dots).

**Table S1. Z-scores as indications of mean deviations between observations and model prediction.**

| Date | Z-score |
|---|---|
| 1/17/2024 | -1.21431 |
| 1/19/2024 | -2.92622 |
| 1/20/2024 | -2.52725 |
| 1/21/2024 | -2.3184 |
| 1/22/2024 | -1.91702 |
| 1/23/2024 | -2.41363 |
| 1/24/2024 | -2.7368 |
| 1/25/2024 | -2.36995 |
| 1/26/2024 | -2.15492 |
| 1/29/2024 | -2.65067 |
| 1/31/2024 | -1.69367 |
| 2/2/2024 | -0.77079 |
| 2/5/2024 | -2.22128 |
| 2/7/2024 | -0.65959 |
| 2/9/2024 | -0.799 |
| 2/12/2024 | 0.131623 |
| 2/14/2024 | -0.38986 |
| 2/16/2024 | -0.39598 |
| 2/19/2024 | 0.241958 |
| 2/21/2024 | -0.43901 |
| 2/23/2024 | -0.70189 |
| 2/26/2024 | -0.59435 |
| 2/28/2024 | 1.85292 |
| 2/29/2024 | 2.798228 |
| 3/1/2024 | 2.384596 |
| 3/2/2024 | 2.008755 |
| 3/3/2024 | 1.632506 |
| 3/4/2024 | 1.007193 |
| 3/6/2024 | 0.750683 |
| 3/8/2024 | 0.914476 |
| 3/11/2024 | 1.006881 |
| 3/13/2024 | -0.09178 |
| 3/18/2024 | 0.187925 |
| 3/20/2024 | -0.21916 |
| 3/22/2024 | 0.158972 |
| 3/27/2024 | -0.47208 |
| 3/28/2024 | 0.262797 |
